# Research Preparedness Among Osteopathic Medical Students in the Post-Pass/Fail Licensing Examination Era: Bridging the Gap Between Rising Expectations and Student Confidence

**DOI:** 10.7759/cureus.116244

**Published:** 2026-09-14

**Authors:** Abbigail Niewchas, Taylor Clark, Jonathan Swiastyn, Diana Hamdan, Christopher Stewart, Magalie Zoungrana, John R Dobson

**Affiliations:** 1 Medical School, College of Osteopathic Medicine, Kansas City University, Joplin, USA; 2 Medical School, University of Kansas Medical Center School of Medicine, Wichita, USA; 3 Medical School, University of Kansas Medical Center School of Medicine, Kansas City, USA; 4 Pathology, College of Osteopathic Medicine, Kansas City University, Kansas City, USA

**Keywords:** medical student attitudes, osteopathic medical schools, research confidence, residency preparation, undergraduate medical student

## Abstract

Background: Since the transition of COMLEX Level 1 and USMLE Step 1 to pass/fail scoring, research productivity has become an increasingly important component of residency applications. National match data demonstrate increasing expectations for research experiences, abstracts, presentations, and publications across specialties. However, limited data describe how osteopathic medical students perceive their research preparedness, access to institutional research resources, and confidence in achieving competitive scholarly productivity in the post-pass/fail licensing examination era.

Methods: A cross-sectional survey of osteopathic medical students assessed research preparedness, institutional support, perceived barriers, and preferred strategies to increase research involvement. Descriptive statistics and exploratory correlation analyses were performed.

Results: A total of 136 osteopathic medical students completed the survey. Respondents expressed strong interest in participating in additional research (mean Likert score, 3.65/5), yet relatively few reported confidence initiating (13.6%), writing (25.8%), or publishing (17.1%) research. Confidence across these domains demonstrated good internal consistency and was combined into a composite ‘Research Confidence’ score for exploratory analyses. Greater prior research experience, scholarly output, and perceived institutional support were each positively associated with Research Confidence. The most frequently reported barriers were limited knowledge of project initiation, time constraints related to course load, and limited access to specialty-specific mentors. Nearly all respondents expressed interest in structured mentorship programs, whereas substantially fewer favored formal research coursework.

Conclusions: Osteopathic medical students report strong research interest but identify substantial institutional and structural barriers associated with lower perceived research preparedness and confidence. Greater research engagement and perceived institutional support were associated with higher Research Confidence, suggesting that mentorship and institutional research infrastructure may play important roles in preparing students for scholarly activity. These findings provide preliminary evidence to inform targeted institutional interventions and future multi-institutional studies examining research preparedness in the post-pass/fail licensing examination era.

## Introduction

Since 2022, the United States Medical Licensing Examination (USMLE) Step 1 and the Comprehensive Osteopathic Medical Licensing Examination (COMLEX) Level 1 have transitioned from numeric scoring to a pass/fail grading system. Before this change, residency program directors commonly used both Step 1/Level 1 and Step 2/Level 2 scores to screen applicants for interviews and to inform rank-order decisions. In the absence of numeric Level 1/Step 1 scores, greater emphasis has been placed on Level 2/Step 2 performance and other aspects of residency applications, including scholarly productivity and extracurricular involvement. Recent surveys of residency program directors suggest that research productivity is increasingly considered in applicant evaluation and is expected to remain an important factor in future selection decisions [1-3]. Consistent with these trends, Radulovich et al. (2023) found that most medical students believed research would become a more heavily weighted component of residency applications following the transition to pass/fail Step 1, although fewer than half reported increasing their research efforts [4]. These findings suggest that increased awareness of research expectations may not translate into greater research participation, highlighting the need to better understand whether students feel adequately prepared to engage in scholarly activity and achieve the scholarly productivity increasingly expected during residency selection.

Data from the National Resident Matching Program (NRMP) and prior studies demonstrate that applicants who successfully match report greater research activity, including abstracts, presentations, and publications, than unmatched applicants across a wide range of specialties [5]. Similarly, nearly all surveyed osteopathic medical students perceive research as an important factor in residency competitiveness and identify it as a primary benefit of research participation [6]. Consistent with these perceptions, Currie et al. (2024) found that students taking Step 1 under the pass/fail system believed substantially more research experiences were necessary to match into their preferred specialty than students who previously received a numeric score [7]. Despite these increasing expectations, access to research opportunities and institutional support varies considerably across medical schools, and barriers such as limited mentorship, funding, and protected time may hinder meaningful research participation [8]. Although students increasingly recognize the importance of research for residency competitiveness, it remains unclear whether their confidence in navigating the research process, access to mentorship, and institutional support have evolved to adequately prepare them to meet these changing expectations.

Research preparedness is influenced by multiple institutional and individual factors, including faculty mentorship, funding opportunities, protected research time, and research infrastructure [9-11]. These disparities may be particularly relevant within osteopathic medical education, where the availability of research resources has been reported to vary across institutions and remains incompletely characterized. However, little is known about how osteopathic medical students perceive their access to these resources or whether these perceptions influence their confidence in achieving the scholarly productivity expected for their intended specialty. Collectively, these factors contribute to research preparedness, which encompasses the confidence, knowledge, mentorship, and institutional resources necessary for students to successfully initiate, conduct, and disseminate scholarly work during medical school.

The objectives of this study were to (1) assess osteopathic medical students' perceptions of institutional research support and their confidence in conducting and publishing scholarly work in the post-Level 1/Step 1 pass/fail era; (2) identify barriers to research involvement; and (3) determine which institutional resources students believe would most improve their research preparedness and participation in scholarly activity. This single-institution survey was designed to evaluate whether a gap exists between the increasing emphasis placed on scholarly productivity in residency selection and osteopathic medical students' perceived preparedness to successfully participate in research.

## Materials and methods

Study design and participants

This exploratory cross-sectional survey study was conducted at Kansas City University College of Osteopathic Medicine (KCU-COM). The electronic survey was distributed to all 1,731 actively enrolled first- through fourth-year osteopathic medical students (OMS-I through OMS-IV). Participation was voluntary, anonymous, and uncompensated.

Survey development

A 23-item electronic survey was developed following a review of the literature and national residency match resources to align survey items with the study objectives and the multidimensional concept of research preparedness. The survey evaluated seven domains related to research preparedness: (1) awareness of specialty-specific research expectations based on national match data; (2) desire to participate in research; (3) confidence in initiating, writing, and publishing research using institutional resources; (4) perceived adequacy of institutional support to achieve competitive research productivity for the student’s intended specialty; (5) availability and perceived usefulness of institutional research resources; (6) perceived barriers to research involvement; and (7) familiarity with and interest in third-party research databases and tools. The present analysis focused on domains directly related to perceived research preparedness, institutional support, barriers to research involvement, and preferred strategies for increasing research participation; items assessing awareness of specialty-specific research expectations and familiarity with third-party research tools were considered exploratory survey domains.

Survey items consisted of multiple-choice questions, select-all-that-apply items, and six-point Likert-scale statements ranging from 0 ("strongly disagree") to 5 ("strongly agree"). Intermediate response options were 1 ("disagree"), 2 ("neutral/disagree"), 3 ("neutral/agree"), and 4 ("agree"). Demographic variables collected included class year, intended specialty category (primary care, non-surgical specialty, surgical specialty, or undecided), self-reported number of prior research experiences, and number of scholarly outputs (publications and/or presentations). Faculty investigators reviewed the survey for content and clarity before distribution, and it was designed to take approximately 10 minutes to complete. An overview of survey domains is provided in Appendix 1, and the complete 23-item survey instrument, including response options, is provided in Appendix 2. 

Survey administration

The anonymous electronic survey was distributed via institutional email listserv to all eligible students. Recruitment emails were sent periodically between May 2025 and January 2026, with reminder emails distributed throughout the recruitment period. First-year students began recruitment later than upper-class students to allow time for exposure to research opportunities before survey completion. Recruitment emails described the study objectives and emphasized that participation was voluntary and uncompensated. Completion of the survey was considered to imply informed consent. The survey remained open throughout the recruitment period, after which responses were exported for analysis.

Outcomes

Consistent with the study framework, perceived research preparedness was assessed through confidence across three stages of the research process: project initiation, manuscript preparation, and publication. The primary outcome was self-reported confidence in initiating a research project, writing a research manuscript, and publishing scholarly work using available institutional resources. Secondary outcomes included perceived adequacy of institutional support to achieve a competitive level of research productivity for the student's intended specialty, desire to participate in research, perceived availability and usefulness of institutional research resources, perceived barriers to research involvement, and interest in structured research support programs. Awareness of specialty-specific research expectations and familiarity with and interest in third-party research databases were assessed as exploratory survey domains but were not included in the primary analyses reported in this manuscript. Covariates included class year, intended specialty category, self-reported number of prior research experiences, and number of scholarly outputs.

Statistical analysis

Descriptive statistics were used to summarize participant characteristics and survey responses. Categorical variables were reported as frequencies and percentages, and Likert-scale responses were summarized using measures of central tendency and dispersion as appropriate based on distribution. Missing data were not imputed. Available responses were analyzed for each survey item, resulting in variation in sample size across outcomes. Internal consistency of the three research confidence items assessing confidence in initiating, writing, and publishing research was evaluated using Cronbach's alpha. Based on the observed internal consistency, these items were averaged to create a composite Research Confidence score for exploratory analyses; the score was calculated only for respondents with complete responses to all three component items. Spearman rank-order correlation analyses were performed to examine associations between prior research experience, scholarly output, perceived institutional support, and Research Confidence using available complete observations for the variables included in each analysis. Statistical analyses were performed using jamovi (version 2.6.45.0), and a two-sided significance level of α = 0.05 was used for all inferential tests. 

Ethical considerations

This study was reviewed and approved by the Kansas City University Institutional Review Board (Study protocol 2283833-1). Participation was voluntary, and informed consent was implied through survey completion. All data were collected anonymously and stored securely in accordance with institutional policies.

## Results

Participant characteristics

A total of 152 survey responses were received from 1,731 eligible students. Sixteen responses were excluded because they were duplicate submissions or did not provide class year or intended specialty, resulting in 136 responses included in the final analysis and an overall analytic response rate of 7.9% (136/1,731). Respondents included 34 (25.0%) students from the Class of 2026, 47 (34.6%) from the Class of 2027, 38 (27.9%) from the Class of 2028, and 17 (12.5%) from the Class of 2029. Intended specialty interests were most commonly non-surgical specialty care (52, 38.2%), followed by primary care (31, 22.8%), surgical specialties (27, 19.9%), and students who were undecided (26, 19.1%) (Table 1).

**Table 1:** Respondent demographic and academic characteristics.

Characteristic	n (%)
Class year	
Class of 2026	34 (25.0)
Class of 2027	47 (34.6)
Class of 2028	38 (27.9)
Class of 2029	17 (12.5)
Intended specialty	
Primary care	31 (22.8)
Non-surgical specialty	52 (38.2)
Surgical specialty	27 (19.9)
Undecided	26 (19.1)

Research interest and perceived research preparedness

Overall, respondents expressed a strong desire to become involved in additional research (mean Likert score, 3.65/5), with 62.9% agreeing or strongly agreeing that they wished to participate in more research projects. Despite this strong interest, measures of research preparedness were consistently lower across multiple domains. Only 13.6% of respondents agreed or strongly agreed that they were confident in initiating an independent research project (mean Likert score, 1.99/5), while 25.8% reported confidence in their ability to write a research manuscript and 17.1% reported confidence in publishing a manuscript. Similarly, only 14.7% agreed or strongly agreed that their institution provided sufficient resources and support to help them achieve the level of research productivity expected for their intended specialty. Across all confidence measures and perceptions of institutional support, the proportion of respondents expressing disagreement exceeded those expressing agreement, highlighting a disconnect between students' interest in research and their perceived preparedness to successfully participate in scholarly activity (Figure 1).

**Figure 1:**
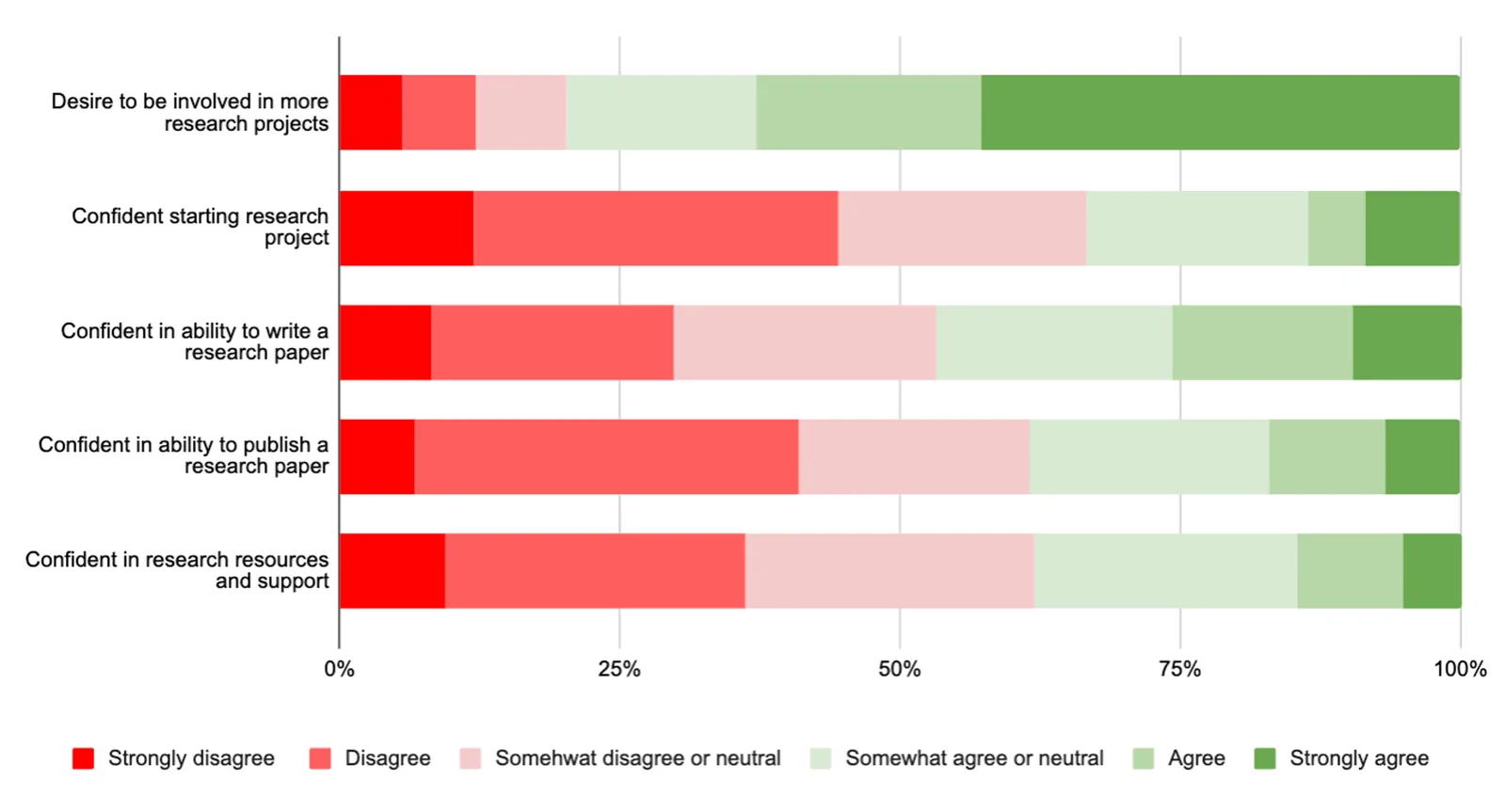
Student interest in research and perceived research preparedness across the research process.

Perceived barriers to research preparedness 

When asked about barriers to research involvement (n = 130), the most frequently identified challenges were limited knowledge of how to initiate a research project and time constraints related to course load, each reported by 76 respondents (58.5%). Barriers related to mentorship and specialty-specific access were also common, including limited access to clinicians within respondents' desired specialty (75, 57.7%) and a lack of faculty advisors to provide research guidance (70, 53.8%). Additional barriers included limited research experience (59, 45.4%) and insufficient funding for publications and conference participation (47, 36.2%). Only 21 respondents (16.2%) indicated that they did not experience these barriers or were not interested in becoming more involved in research (Figure 2).

**Figure 2:**
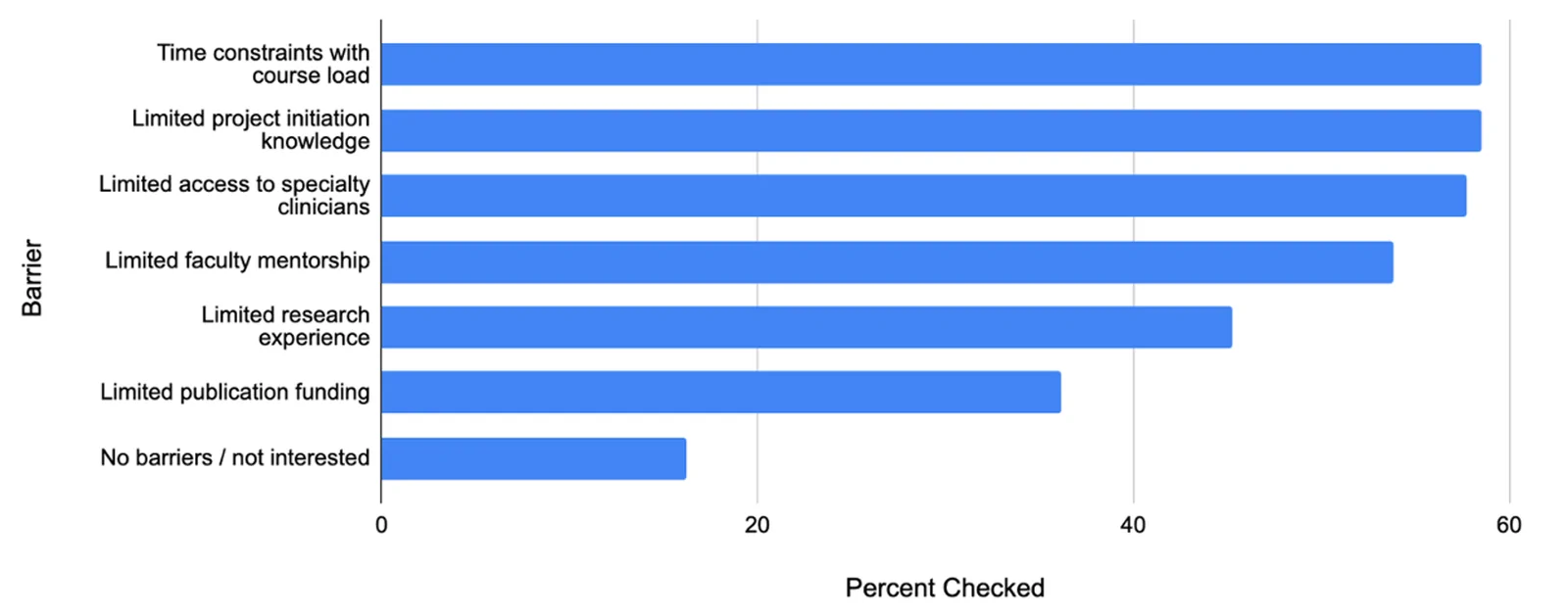
Perceived barriers to research involvement among survey respondents (n=130).

Student-preferred strategies to improve research preparedness

Among respondents who answered these questions (n = 134), interest in structured research support was high. The most frequently endorsed intervention was a mentorship program pairing students with specialty-specific residents and attending physicians to facilitate research participation (125, 93.3%). Interest was similarly high in a faculty mentorship program matching students with specialty-specific faculty mentors to guide research initiatives (119, 88.8%). In comparison, interest in a structured research methodology course was lower, although more than half of respondents indicated they would participate (77, 57.5%). A summary of the percentage of students interested in specific strategies to improve research preparedness can be found in Figure 3 below.

**Figure 3:**
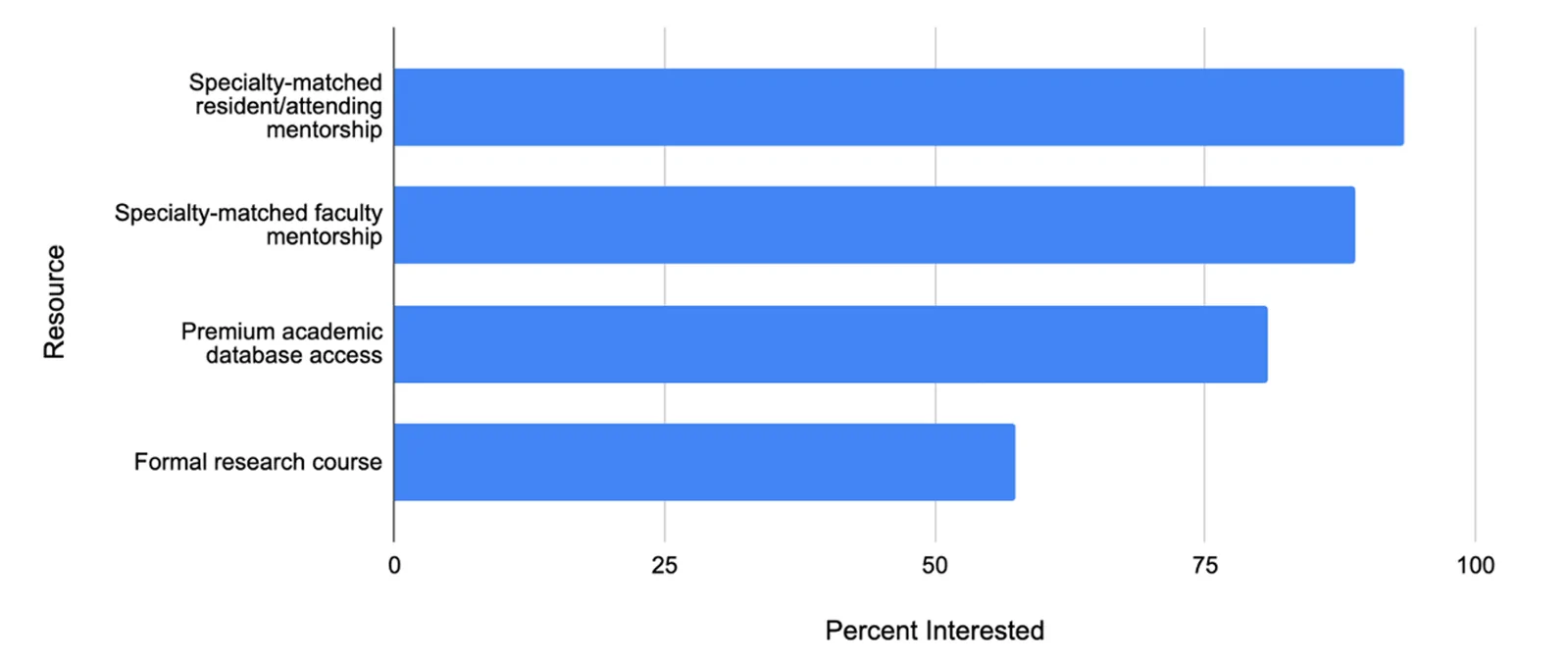
Percentage of respondents expressing interest in institutional resources designed to increase research involvement (n = 134).

Among respondents who completed the resource-ranking question (n = 132), mentorship from specialty-specific physicians was identified as the most helpful resource for improving research preparedness and increasing research involvement (55, 41.7%), followed by dedicated time for research (33, 25.0%) and mentorship from faculty based on students' specialty interests (30, 22.7%) (Figure 4). 

**Figure 4:**
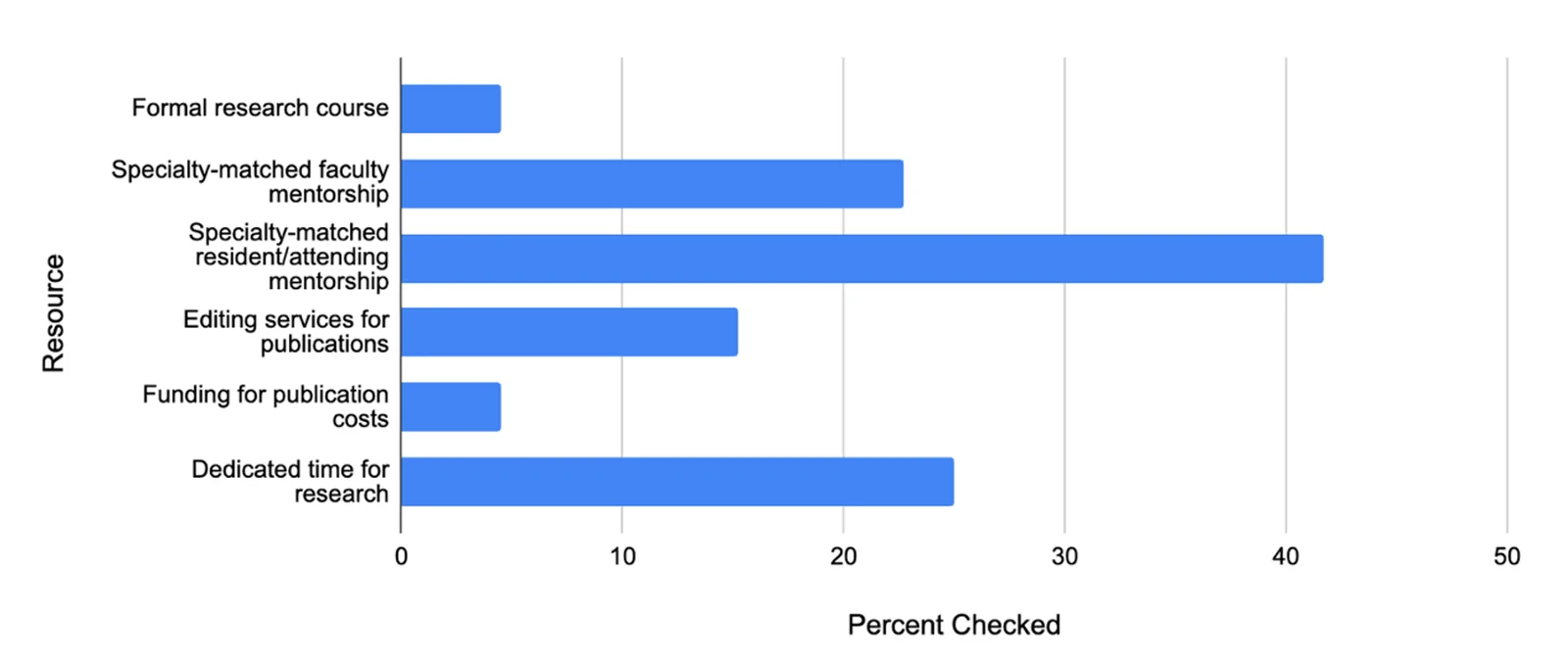
Student-ranked resources to increase research involvement. Percentage of respondents identifying the single most helpful institutional resource for increasing their research involvement (n = 132).

Factors associated with research confidence

The three survey items assessing confidence initiating, writing, and publishing research demonstrated good internal consistency (Cronbach's α = 0.892). These items were therefore averaged to create a composite ‘Research Confidence’ score. Among respondents with complete data for all three confidence items (n = 110), the mean Research Confidence score was 2.28 ± 1.27 (possible range, 0-5). Greater prior research experience (Spearman's ρ = 0.233, p = 0.014) and prior scholarly output (Spearman's ρ = 0.278, p = 0.003) were positively associated with Research Confidence. Perceived institutional support demonstrated the strongest association with Research Confidence (Spearman's ρ = 0.570, p < 0.001) (Table 2).

**Table 2:** Factors associated with Research Confidence among osteopathic medical students (n=110).

Predictor	Outcome	Spearman's ρ	p value
Prior research experience	Research Confidence	0.233	0.014
Prior scholarly output	Research Confidence	0.278	0.003
Perceived institutional support	Research Confidence	0.570	<0.001

## Discussion

This study evaluated osteopathic medical students' perceptions of research preparedness, institutional support, and barriers to research involvement in the post-pass/fail licensing examination era. Three principal findings emerged. First, respondents expressed a strong desire to participate in research but reported low confidence in initiating and publishing scholarly work. Second, students identified mentorship, time constraints, and limited knowledge of project initiation as the primary barriers to research involvement. Third, mentorship-based programs were overwhelmingly favored over structured research coursework as potential approaches to supporting research participation. These findings are consistent with an emerging body of post-pass/fail literature. Multiple studies have demonstrated that medical students increasingly perceive research as an important component of residency applications following the transition to pass/fail licensing examinations [4,7]. Within this context, our findings demonstrate a gap between respondents' strong interest in research and their perceived preparedness to participate in scholarly activity. This gap may be particularly relevant within osteopathic medical education, where comparatively fewer research resources, lower NIH funding, and fewer structured research opportunities have historically limited scholarly productivity relative to many allopathic institutions [12-14].

Although previous studies have shown that medical students increasingly perceive research as important following the transition to pass/fail licensing examinations, relatively few students report increasing their research involvement [4]. Our findings identify several structural barriers that may contribute to this discrepancy, including limited mentorship, uncertainty surrounding project initiation, and time constraints with existing course load. While the present study cannot determine whether these barriers are attributable to the transition of licensing exams to pass/fail, their presence despite strong student interest in research involvement suggests that institutional factors may influence students' ability to translate research interest into participation. These findings are consistent with previous studies conducted both before and after the transition of USMLE Step 1 and COMLEX Level 1 to pass/fail scoring, which similarly identified mentorship, protected time, and difficulty navigating research opportunities as persistent barriers to student scholarly activity [8,15,16]. The persistence of these barriers across studies highlights mentorship, research infrastructure, and access to opportunities as areas warranting further investigation as institutions seek to support student scholarly participation.

One notable finding was the substantially greater student interest in mentorship-based programs compared with formal research coursework. Although structured research courses may improve knowledge of study design and scientific writing, respondents appeared to prioritize individualized guidance and direct access to active investigators. This preference may reflect the practical challenges identified elsewhere in the survey, particularly uncertainty regarding how to initiate projects and limited access to specialty-specific mentors. This notion is supported by Shen et al. (2026), who reported favorable outcomes following implementation of a specialty-specific mentor-matched research program, with most participating students rating the quality of mentorship, research projects, and their overall experience as moderately to extremely positive [17]. Rather than functioning solely as research advisors, mentors may bridge multiple components of research preparedness by helping students identify projects, navigate project initiation, develop scholarly writing skills, and establish specialty-specific professional networks. These potential roles align with several of the barriers identified in our survey and may help explain respondents' preference for mentorship over additional didactic instruction. 

Perhaps the most striking finding was the disconnect between students’ interest in greater research involvement and their confidence in conducting research. Nearly two-thirds of respondents expressed a desire to become more involved in research, yet fewer than one in six felt confident initiating an independent research project. Furthermore, greater prior research experience, scholarly output, and perceived institutional support were all associated with higher Research Confidence, with institutional support demonstrating the strongest association. Although these cross-sectional findings cannot establish causality, they suggest that students who perceive stronger institutional support also report greater confidence navigating the research process. Similar concerns have been reported elsewhere following implementation of pass/fail Level 1 and Step 1. Rothka et al. (2024) found that students reported increased anxiety surrounding Step 2 performance and emphasized the need for additional mentorship and guidance regarding residency preparation [18]. These findings provide broader context for the challenges reported by students in the present study but do not establish that the transition to pass/fail examinations caused the observed differences in research confidence or perceived preparedness. Rather, our findings characterize these perceptions among osteopathic medical students navigating residency preparation in the contemporary post-pass/fail environment.

As residency programs increasingly adopt holistic applicant review processes, ensuring equitable access to research mentorship and meaningful research opportunities may become increasingly important [19]. Although medical schools cannot directly influence evolving residency selection expectations, our findings suggest that institutional support is closely associated with students' confidence in navigating the research process. Expanding mentorship networks, facilitating early exposure to research, and reducing barriers to project initiation may therefore represent promising institutional strategies warranting prospective evaluation. These strategies may be particularly important for osteopathic medical schools, where national survey data demonstrate substantial variability in research infrastructure, structured research programs, and institutional support for student scholarly activity [14]. 

Limitations

This study has several limitations. First, the survey instrument was developed specifically for this study and, although reviewed by faculty investigators for content and clarity, was not formally pilot tested or psychometrically validated prior to administration. Although the three items comprising the Research Confidence score demonstrated good internal consistency, this does not establish the validity of the composite as a measure of the broader construct of research preparedness. Second, the study was conducted at a single osteopathic medical institution, which may limit the generalizability of the findings to other osteopathic or allopathic medical schools. Although the survey included items intended to facilitate future comparisons between MD and DO students, only osteopathic medical students were included in the present analysis.

Participation was voluntary, and the analytic response rate was 7.9%, introducing the potential for selection and nonresponse bias. Students with greater interest in research or stronger perceptions regarding institutional research support may have been more likely to participate, and respondents may therefore not be representative of the broader eligible student population. In addition, the study relied on self-reported measures of research experience, scholarly output, confidence, and institutional support, which are subject to recall and social desirability bias. No objective measure of research preparedness or independently verified measure of scholarly productivity was available to validate respondents' perceptions or reported research activity. Because both the factors examined in the correlation analyses and Research Confidence were collected through the same self-report survey, the observed associations may also be influenced by common-method bias.

Students at different stages of training completed the survey after varying levels of exposure to institutional research opportunities, which may have influenced their perceptions of research preparedness and institutional support. Although students from all four class years and multiple specialty-interest categories were represented, subgroup sizes were relatively small and uneven, limiting more granular comparisons across class years and intended specialties. Finally, the cross-sectional design prevents assessment of causal or temporal relationships. Accordingly, the observed associations between research experience, scholarly output, institutional support, and Research Confidence should be interpreted as correlational rather than causal.

Despite these limitations, this exploratory study provides preliminary evidence regarding osteopathic medical students' perceived research preparedness and identifies several areas for further investigation, including mentorship, early research engagement, and support for project initiation.

## Conclusions

Osteopathic medical students demonstrated strong interest in research participation but reported limited confidence in their ability to initiate and publish scholarly work, highlighting a disconnect between increasing expectations for scholarly productivity and students' perceived research preparedness in the post-pass/fail licensing examination era. The most commonly identified challenges included limited knowledge of how to initiate a research project, limited access to specialty-specific mentors, and time constraints related to course load. Greater prior research experience, scholarly output, and perceived institutional support were each associated with higher Research Confidence, with institutional support demonstrating the strongest association. Students overwhelmingly favored mentorship-based research programs over formal research coursework, suggesting that structured mentorship may represent a promising institutional strategy to address perceived barriers to research participation and warrants prospective evaluation. Collectively, these findings characterize perceived research preparedness among osteopathic medical students in the post-pass/fail licensing examination era and identify institutional support and mentorship as areas warranting further investigation. Future studies should evaluate whether structured mentorship and other institutional interventions are associated with improved Research Confidence, scholarly productivity, and research preparedness across multiple and diverse medical education settings.

## Appendices

Appendix 1

**Table 3:** Survey domains, survey items, and response format.

Survey Domain	Survey Item (Abbreviated Wording)	Response Format	
Knowledge of Match Expectations	Awareness of average number of research activities required for successful match	Multiple choice	
Knowledge Sources	Resources used to obtain match statistics (NRMP [20], AAMC [21], etc.)	Select all that apply	
Desire for Research	Desire to be involved in additional research	6-point Likert scale	
Specialty Interest	Intended specialty category (primary care, non-surgical specialty, surgical specialty, undecided)	Multiple choice	
Research Confidence	Confidence in initiating a research project	6-point Likert scale	
Research Confidence	Confidence in writing a research manuscript	6-point Likert scale	
Research Confidence	Confidence in publishing a research manuscript	6-point Likert scale	
Competitive Readiness	Confidence that institutional resources allow achievement of specialty-specific research expectations	6-point Likert scale	
Priority Resource	Single most helpful resource to increase research involvement	Single choice	
Institutional Infrastructure	Presence of dedicated research staff	Multiple choice	
Institutional Infrastructure	Presence of an office/department of research	Multiple choice	
Institutional Support	Coverage of publication and/or conference costs	Multiple choice	
Research Resources	Availability of specific research supports (courses, mentorship, funding, protected time)	Select all that apply	
Barriers	Perceived barriers to research involvement	Select all that apply	
Program Interest	Interest in structured research programs (faculty mentorship, research courses, resident/attending partnerships)	Multiple choice	
Third-Party Tools	Familiarity with Consensus [22], CMS Data [23], TriNetX [24]	Select all that apply	
Third-Party Tools	Willingness to learn/use third-party databases if provided	Multiple choice	
Demographics	Degree program (MD vs DO)	Multiple choice	
Demographics	Class year	Multiple choice	
Research Experience	Number of prior research experiences	Ordinal categories	
Scholarly Output	Number of publications and/or presentations	Ordinal categories	

Appendix 2 

**Table 4:** Complete survey instrument and response options. Questions 8–12 were assessed using a six-point Likert scale ranging from 0 ("strongly disagree") to 5 ("strongly agree"). Questions 7, 16, and 17 permitted selection of multiple responses. Additional survey information: For Q4, respondents were provided examples of research experiences encompassing basic science, clinical/translational science, medical education, public health, and quality improvement, with a link to the AAMC ERAS Experiences resource for additional information. For Q5, respondents were provided examples of ERAS publication categories, including peer-reviewed journal articles/abstracts, articles/abstracts other than published, book chapters, scientific monographs, poster presentations, oral presentations, and peer- and non-peer-reviewed online publications. For Q6, respondents were provided links to AAMC residency data and NRMP Match Data. For Q21, respondents were provided descriptions and links for Consensus, CMS Data, and TriNetX.

Question	Survey item	Response options
Q1	Please select the degree you are working towards.	MD; DO
Q2	Class year	2026; 2027; 2028; 2029
Q3	Top choice for specialty	Primary care; Specialty care (non-surgical); Surgical specialty; Undecided
Q4	How many research experiences do you currently have? Examples of primary focus areas for research experiences were provided for basic science, clinical/translational science, medical education, public health, and quality improvement. Respondents were also provided a link to the AAMC ERAS Experiences resource for additional information [25].	0; 1-4; 5-9; 10-15; >15
Q5	How many publications and/or presentations do you currently have? ERAS publication categories were provided, including peer-reviewed journal articles/abstracts, peer-reviewed journal articles/abstracts other than published, peer-reviewed book chapters, scientific monographs, poster presentations, oral presentations, peer-reviewed online publications, and non-peer-reviewed online publications.	0; 1-5; 6-10; 11-15; 16-20; >20
Q6	Do you know (approximately) the average number of research activities and/or publications/presentations that successful 4th-year medical students obtained in your desired specialty to achieve a successful match this year?	Yes; No; Undecided on specialty – aware of average number for specialties I am interested in; Undecided on specialty – not aware of average number for specialties I am interested in
Q7	Which of the following resources have you utilized to obtain information on match statistics?	NRMP [20]; AAMC [21]; Other news source (e.g., Med Central, PR Newswire); Other social media platform (e.g., Twitter, Reddit); I have not utilized any of the above resources to obtain information on match statistics
Q8	I have a desire to be involved in more research projects (including starting my own, working with others, etc.).	0 = Strongly disagree; 1 = Disagree; 2 = Neutral/disagree; 3 = Neutral/agree; 4 = Agree; 5 = Strongly agree
Q9	I feel confident in starting my own research project with the resources available through my institution.	0 = Strongly disagree; 1 = Disagree; 2 = Neutral/disagree; 3 = Neutral/agree; 4 = Agree; 5 = Strongly agree
Q10	I feel confident in my ability to write a research paper for publication with the resources available through my institution.	0 = Strongly disagree; 1 = Disagree; 2 = Neutral/disagree; 3 = Neutral/agree; 4 = Agree; 5 = Strongly agree
Q11	I feel confident in my ability to publish a research manuscript with the resources available through my institution.	0 = Strongly disagree; 1 = Disagree; 2 = Neutral/disagree; 3 = Neutral/agree; 4 = Agree; 5 = Strongly agree
Q12	I feel that my institution has the resources and support for me to achieve the average number of research activities and/or publications and presentations for me to match into my desired specialty.	0 = Strongly disagree; 1 = Disagree; 2 = Neutral/disagree; 3 = Neutral/agree; 4 = Agree; 5 = Strongly agree
Q13	Does your institution have a full-time employee(s) dedicated to facilitating research for medical students? (e.g., research facilitator or coordinator)	Yes; No; I don't know
Q14	Does your institution have an office of research or a department of research designed to provide support for medical students conducting research?	Yes; No; I don't know
Q15	Does your institution provide funding for the publication of research projects and/or to cover the cost of presenting research at scientific conferences?	Yes; No; Other
Q16	Does your institution possess any of the following? Select all that apply.	Introduction to research course (required or elective); Research mentorship (faculty); Research mentorship/partnership (residents/attendings); Editing services for publications; Funding for publications and conference presentations; Dedicated time for research
Q17	Overall, if you feel that you are unable to obtain a competitive amount of research for your desired specialty, what barriers are preventing you from doing so, or from participating in more research than what is available to you? Select all that apply.	Limited experience in research; Limited knowledge of how to start a research project through my institution; Lack of funding opportunities for publications and conferences; Lack of faculty advisors for guidance of the research process; Lack of access to clinicians (residents/attendings) in my desired specialty; Time constraints with course load; I don't want to be involved in more research/do not feel I face these barriers to increase my research involvement
Q18	Suppose your institution created a formal program that matched medical students with faculty mentors (based on specialty interest) to assist in the review of research initiatives. Would you be interested in participating?	Yes; No; This program exists at my institution and I utilize it or have done so in the past; This program exists at my institution, but I do not utilize it
Q19	Suppose your institution created a course aimed at educating medical students on how to create their own research project, how to obtain and analyze data, and assist with scientific writing for publication. Would you be interested in enrolling? Assume time commitment is 1–2 hours/week.	Yes; No; This program exists at my institution and I utilize it or have done so in the past; This program exists at my institution, but I do not utilize it
Q20	Suppose your institution created a formal program that allowed medical students to match with residents and attendings to conduct research in their field of practice/interest. Would you be interested in participating?	Yes; No; This program exists at my institution and I utilize it or have done so in the past; This program exists at my institution, but I do not utilize it
Q21	Are you familiar with databases and academic search engines such as Consensus [22], CMS Data [23], or TriNetX [24]? Brief descriptions of each resource were provided to respondents.	Yes; No; Somewhat/have heard of some but not all
Q22	Suppose your institution purchased premium access to one or more of the aforementioned search engines/databases (Consensus [22], CMS Data [23], and TriNetX [24]). Would you be willing to learn how to navigate them in order to utilize them to conduct your own research?	Yes; No; This program exists at my institution and I utilize it or have done so in the past; This program exists at my institution, but I do not utilize it
Q23	Overall, what would be the single most helpful resource for you to increase your research involvement? If you feel that you are adequately supported by your institution for your research endeavors, please skip this question.	Research course; Mentoring from faculty based on specialty interest; Mentoring/partnership with residents/attendings in desired field; Editing services for publications; Funding for publications and conferences; Dedicated time for research; Access to academic search engines and patient databases
